# Exponential operations and aggregation operators of interval neutrosophic sets and their decision making methods

**DOI:** 10.1186/s40064-016-3143-z

**Published:** 2016-09-05

**Authors:** Jun Ye

**Affiliations:** Department of Electrical and Information Engineering, Shaoxing University, 508 Huancheng West Road, Shaoxing, 312000 Zhejiang Province People’s Republic of China

**Keywords:** Interval neutrosophic set, Exponential operation, Interval neutrosophic weighted exponential aggregation (INWEA) operator, Dual interval neutrosophic weighted exponential aggregation (DINWEA) operator, Decision making

## Abstract

An interval neutrosophic set (INS) is a subclass of a neutrosophic set and a generalization of an interval-valued intuitionistic fuzzy set, and then the characteristics of INS are independently described by the interval numbers of its truth-membership, indeterminacy-membership, and falsity-membership degrees. However, the exponential parameters (weights) of all the existing exponential operational laws of INSs and the corresponding exponential aggregation operators are crisp values in interval neutrosophic decision making problems. As a supplement, this paper firstly introduces new exponential operational laws of INSs, where the bases are crisp values or interval numbers and the exponents are interval neutrosophic numbers (INNs), which are basic elements in INSs. Then, we propose an interval neutrosophic weighted exponential aggregation (INWEA) operator and a dual interval neutrosophic weighted exponential aggregation (DINWEA) operator based on these exponential operational laws and introduce comparative methods based on cosine measure functions for INNs and dual INNs. Further, we develop decision-making methods based on the INWEA and DINWEA operators. Finally, a practical example on the selecting problem of global suppliers is provided to illustrate the applicability and rationality of the proposed methods.

## Background

Neutrosophic sets and single-valued neutrosophic sets (SVNSs) were introduced for the first time by Smarandache (1998). Then, interval neutrosophic sets (INSs) were presented by Wang et al. (2005). However, SVNSs and INSs are the subclasses of neutrosophic sets and the generalization of intuitionistic fuzzy sets (IFSs) and interval-valued intuitionistic fuzzy sets (IVIFSs). The characteristics of SVNS and INS are described independently by the truth-membership, indeterminacy-membership, and falsity-membership degrees. The main advantage of the neutrosophic set is that it is a powerful general formal framework for expressing and handling incomplete, indeterminate, and inconsistent information, which exists in real situations; while IFSs and IVIFSs cannot express and deal with the indeterminate and inconsistent information. Recently, many researchers have presented various algorithms and applications of SVNSs and INSs (Guo et al. 2014; Ye 2014a, 2015a, b; Peng et al. 2014, 2015; Majumdar and Samant 2014; Sahin and Kucuk 2014; Zhang et al. 2015; Biswas et al. 2015). Then, some basic operations on SVNSs and INSs, such as addition, exponent, and multiplication, and some corresponding aggregation operators have been introduced recently. For instance, Wang et al. (2005, 2010) introduced some basic operations of SVNSs and INSs. Ye (2014b) defined some basic operational laws of simplified neutrosophic sets (SNSs), which include SVNSs and INSs, and proposed some weighted averaging aggregation operators to aggregate simplified neutrosophic information, and then applied these aggregation operators to multiple attribute decision making (MADM). Then, Zhang et al. (2014) pointed out the drawbacks of some operational laws of SNSs and improved some operational laws of INSs and some aggregation operators of INSs, and then applied them to MADM problems with interval neutrosophic information. Further, Liu and Wang (2014) proposed single-valued neutrosophic normalized weighted Bonferroni mean operators and applied them to MADM problems. Also, Liu et al. (2014) introduced some generalized neutrosophic number Hamacher aggregation operators and applied them to multiple attribute group decision making problems. Ye (2015c) proposed interval neutrosophic ordered weighted arithmetic and geometric averaging operators and the possibility degree ranking method and applied them to MADM problems under an interval neutrosophic environment. Zhao et al. (2015) developed an interval neutrosophic generalized weighted aggregation operator for MADM. Furthermore, Ye (2015d) put foreword interval neutrosophic weighted arithmetic and geometric averaging operators with credibility information and applied them to interval neutrosophic MADM problems with credibility information. Liu and Li (2015) introduced a MADM method based on some normal neutrosophic Bonferroni mean operators. Liu and Teng (2015) proposed a MADM method based on a normal neutrosophic generalized weighted power averaging operator. Liu and Tang (2016) presented some power generalized aggregation operators of the interval neutrosophic numbers for decision making. Liu and Wang (2016) developed an interval neutrosophic prioritized ordered weighted average operator and its application in MADM.

However, it should be noted that in the existing literature the basic elements in the single-valued and interval neutrosophic weighted geometric operators consist of crisp values (weights) and SVNSs or INSs. Recently, Gou et al. (2015a) defined exponential operational laws of IFSs, where the bases are crisp values and the exponents are IFSs, and presented an intuitionistic fuzzy exponential aggregation operator with crisp parameters and its application in MADM problems. Further, Gou et al. (2015b) defined exponential operational laws of IVIFSs, where the bases are crisp values or interval numbers and the exponents are IVIFSs, and presented the corresponding exponential aggregation operators and their applications in MADM problems. In the existing decision making problems with single-valued neutrosophic information and interval neutrosophic information, the exponential values (weights) of all the existing exponential operational laws of SVNSs and INSs and the corresponding aggregation operators are positive real numbers within the unit interval [0, 1]. Whereas, all the existing exponential operations and aggregation methods cannot handle such an issue where the bases are crisp values or interval numbers and the exponents are SVNSs and INSs. Hence, we lack some important operational laws and aggregation operators with crisp parameters or interval-valued parameters by using SVNSs or INSs as exponents to handle the decision making problems. Motivated by the exponential operational laws of IFSs and IVIFSs and the corresponding exponential aggregation methods (Gou et al. 2015a, b), it is necessary that we should extend the existing exponential operations of IFSs and IVIFSs to the exponential operational laws of SVNSs and INSs with crisp parameters and interval-valued parameters and propose the corresponding exponential aggregation operators as an important supplement of the existing simplified neutrosophic aggregation techniques. Since INSs reduce to SVNSs when upper and lower ends in interval numbers are equal, this paper only defines the exponential operational laws of INSs with crisp parameters and interval-valued parameters, where the bases are crisp values and interval numbers and the exponents are INSs, and proposes an interval neutrosophic weighted exponential aggregation (INWEA) operator, a dual interval neutrosophic weighted exponential aggregation (DINWEA) operator, and comparative methods for interval neutrosophic numbers (INNs) and dual interval neutrosophic numbers (DINNs). Then, we develop MADM methods by using the INWEA and DINWEA operators, where the data in the decision matrix are given by using crisp values or interval numbers as the evaluation values of attributes and the attribute weights are provided by INNs (basic elements in INS). Finally, we apply these methods to solve the practical problem of selecting the best global supplier for some manufacturing company.

The rest of this paper is structured as follows: “Some basic knowledge and operations of INSs” section reviews some basic knowledge and operations of INSs. “Exponential operational laws of INSs” section proposes exponential operational laws of INSs with crisp parameters and interval-valued parameters as the extension of the existing exponential operations of IVIFSs (Gou et al. 2015b). “Exponential aggregation operators of INNs” section presents the exponential aggregation operators of INSs based on these exponential operational laws and comparative methods for INNs and DINNs. MADM methods are developed by using the INWEA and DINWEA operators in “Decision making methods based on the INWEA and DINWEA operators” section. In “Practical example and comparative analysis” section, a practical example on the selecting problem of global suppliers is provided to illustrate the application and rationality of the developed methods. Some conclusions and future work are contained in “Conclusion” section.

## Some basic knowledge and operations of INSs

The neutrosophic set introduced from a philosophical point of view is difficult to be applied in practical problems since its truth-membership, indeterminacy-membership, and falsity-membership functions lie in the nonstandard interval ]^−^0, 1^+^[. As a simplified form of the neutrosophic set, Wang et al. (2005) defined an INS when its three functions are restricted in the real standard interval [0, 1].

### **Definition 1**

(Wang et al. 2005). Let *X* be a universe of discourse. An INS *N* in *X* is independently characterized by a truth-membership function *T*_*N*_(*x*), an indeterminacy-membership function *I*_*N*_(*x*), and a falsity-membership function *F*_*N*_(*x*) for each *x* ∈ *X*, where $T_{N}^{{}} (x) = [T_{N}^{L} (x),T_{N}^{U} (x)] \subseteq [0,1]$, $I_{N}^{{}} (x) = [I_{N}^{L} (x),I_{N}^{U} (x)] \subseteq [0,1]$, and $F_{N}^{{}} (x) = [F_{N}^{L} (x),F_{N}^{U} (x)] \subseteq [0,1]$, then they satisfy the condition $0 \le T_{N}^{U} (x) + I_{N}^{U} (x) + F_{N}^{U} (x) \le 3$. Thus, the INS *N* can be denoted as $N = \left\{ {\left\langle {x,[T_{N}^{L} (x),T_{N}^{U} (x)],[I_{N}^{L} (x),I_{N}^{U} (x)],[F_{N}^{L} (x),F_{N}^{U} (x)]} \right\rangle |x \in X} \right\}$.

For convenience, a basic element $\left\langle {x,[T_{N}^{L} (x),T_{N}^{U} (x)],[I_{N}^{L} (x),I_{N}^{U} (x)],[F_{N}^{L} (x),F_{N}^{U} (x)]} \right\rangle$ in an INS *N* is denoted by $a = \left\langle {[T_{a}^{L} ,T_{a}^{U} ],[I_{a}^{L} ,I_{a}^{U} ],[F_{a}^{L} ,F_{a}^{U} ]} \right\rangle$ for short, which is called an INN.

Let $a = \left\langle {[T_{a}^{L} ,T_{a}^{U} ],[I_{a}^{L} ,I_{a}^{U} ],[F_{a}^{L} ,F_{a}^{U} ]} \right\rangle$ and $b = \left\langle {[T_{b}^{L} ,T_{b}^{U} ],[I_{b}^{L} ,I_{b}^{U} ],[F_{b}^{L} ,F_{b}^{U} ]} \right\rangle$ be two INNs, then there are the following relations (Wang et al. 2005; Zhang et al. 2014):

1. $$ a^{c} = \left\langle {[F_{a}^{L} ,F_{a}^{U} ],[1 - I_{a}^{U} ,1 - I_{a}^{L} ],[T_{a}^{L} ,T_{a}^{U} ]} \right\rangle $$ (complement of *a*).
2. *a* ⊆ *b* if and only if $T_{a}^{L} \le T_{b}^{L}$, $T_{a}^{U} \le T_{b}^{U}$ $I_{a}^{L} \ge I_{b}^{L}$, $I_{a}^{U} \ge I_{b}^{U}$, $F_{a}^{L} \ge F_{b}^{L}$, and $F_{a}^{U} \ge F_{b}^{U}$;
3. *a* = *b* if and only if *a* ⊆ *b* and *b* ⊆ *a*;
4. $$ a \oplus b = \left\langle {\left[ {T_{a}^{L} + T_{b}^{L} - T_{a}^{L} T_{b}^{L} ,T_{a}^{U} + T_{b}^{U} - T_{a}^{U} T_{b}^{U} } \right],\;\left[ {I_{a}^{L} I_{b}^{L} ,I_{a}^{U} I_{b}^{U} } \right],\;\left[ {F_{a}^{L} F_{b}^{L} ,F_{a}^{U} F_{b}^{U} } \right]} \right\rangle $$;
5. $$ a \otimes b = \left\langle {\left[ {T_{a}^{L} T_{b}^{L} ,\;T_{a}^{U} T_{b}^{U} } \right],\;\left[ {I_{a}^{L} + I_{b}^{L} - I_{a}^{L} I_{b}^{L} ,\;I_{a}^{U} + I_{b}^{U} - I_{a}^{U} I_{b}^{U} } \right],\;\left[ {F_{a}^{L} + F_{b}^{L} - F_{a}^{L} F_{b}^{L} ,\;F_{a}^{U} + F_{b}^{U} - F_{a}^{U} F_{b}^{U} } \right]} \right\rangle ; $$;
6. $$ \mu a = \left\langle {\left[ {1 - \left( {1 - T_{a}^{L} } \right)^{\mu } ,\;1 - \left( {1 - T_{a}^{U} } \right)^{\mu } } \right],\;\left[ {\left( {I_{a}^{L} } \right)^{\mu } ,\;\left( {I_{a}^{U} } \right)^{\mu } } \right],\left[ {\left( {F_{a}^{L} } \right)^{\mu } ,\;\left( {F_{a}^{U} } \right)^{\mu } } \right]} \right\rangle $$ for μ > 0;
7. $$ a^{\mu } = \left\langle {\left[ {\left( {T_{a}^{L} } \right)^{\mu } ,\left( {T_{a}^{U} } \right)^{\mu } } \right],\;\left[ {1 - \left[ {1 - I_{a}^{L} } \right]^{\mu } ,\;1 - \left( {1 - I_{a}^{U} } \right)^{\mu } } \right],\;\left[ {1 - \left( {1 - F_{a}^{L} } \right)^{\mu } ,\;1 - \left( {1 - F_{a}^{U} } \right)^{\mu } } \right]} \right\rangle $$ for μ > 0.

Let $a_{j} = \left\langle {[T_{{a_{j} }}^{L} ,T_{{a_{j} }}^{U} ],[I_{{a_{j} }}^{L} ,I_{{a_{j} }}^{U} ],[F_{{a_{j} }}^{L} ,F_{{a_{j} }}^{U} ]} \right\rangle$(*j* = 1, 2, …, *n*) be a collection of INNs. Based on the weighted aggregation operators of INNs (Zhang et al. 2014), we can introduce the following interval neutrosophic weighted arithmetic and geometric average operators (Zhang et al. 2014):

1 $$ INWAA(a_{1} ,a_{2} , \ldots ,a_{n} ) = \sum\limits_{j = 1}^{n} {w_{j} a_{j} = } \left\langle {\begin{array}{*{20}l} {\left[ {1 - \prod\limits_{j = 1}^{n} {\left( {1 - T_{{a_{j} }}^{L} } \right)^{{w_{{_{j} }} }} } ,\;1 - \prod\limits_{j = 1}^{n} {\left( {1 - T_{{a_{j} }}^{U} } \right)^{{w_{{_{j} }} }} } } \right],} \hfill \\ {\left[ {\prod\limits_{j = 1}^{n} {\left( {I_{{a_{j} }}^{L} } \right)^{{w_{{_{j} }} }} } ,\;\prod\limits_{j = 1}^{n} {\left( {I_{{a_{j} }}^{U} } \right)^{{w_{{_{j} }} }} } } \right],\;\left[ {\prod\limits_{j = 1}^{n} {\left( {F_{{a_{j} }}^{L} } \right)^{{w_{{_{j} }} }} } ,\;\prod\limits_{j = 1}^{n} {\left( {F_{{a_{j} }}^{U} } \right)^{{w_{{_{j} }} }} } } \right]} \hfill \\ \end{array} } \right\rangle , $$

2 $$ INWGA(a_{1} ,a_{2} , \ldots ,a_{n} ) = \prod\limits_{j = 1}^{n} {a_{j}^{{w_{j} }} } = \left\langle {\begin{array}{*{20}l} {\left[ {\prod\limits_{j = 1}^{n} {\left( {T_{{a_{j} }}^{L} } \right)^{{w_{{_{j} }} }} } ,\;\prod\limits_{j = 1}^{n} {\left( {T_{{a_{j} }}^{U} } \right)^{{w_{{_{j} }} }} } } \right],} \hfill \\ {\left[ {1 - \prod\limits_{j = 1}^{n} {\left( {1 - I_{{a_{j} }}^{L} } \right)^{{w_{{_{j} }} }} } ,\;1 - \prod\limits_{j = 1}^{n} {\left( {1 - I_{{a_{j} }}^{U} } \right)^{{w_{{_{j} }} }} } } \right],} \hfill \\ {\left[ {1 - \prod\limits_{j = 1}^{n} {\left( {1 - F_{{a_{j} }}^{L} } \right)^{{w_{{_{j} }} }} } ,\;1 - \prod\limits_{j = 1}^{n} {\left( {1 - F_{{a_{j} }}^{U} } \right)^{{w_{{_{j} }} }} } } \right]} \hfill \\ \end{array} } \right\rangle , $$

where *w*_*j*_ (*j* = 1, 2, …, *n*) is the weight of *a*_*j*_ (*j* = 1, 2, …, *n*) with *w*_*j*_ ∈ [0, 1] and $\sum\nolimits_{j = 1}^{n} {w_{j} } = 1$.

## Exponential operational laws of INSs

### Exponential operations of INSs with crisp parameters

As an extension of existing operational laws of IVIFSs, we define the new exponential operational law of INSs, where the bases are crisp values and the exponents are INSs.

#### **Definition 2**

Let *X* be a universe of discourse and $N = \left\{ {\left\langle {x,[T_{N}^{L} (x),T_{N}^{U} (x)],[I_{N}^{L} (x),I_{N}^{U} (x)],[F_{N}^{L} (x),F_{N}^{U} (x)]} \right\rangle |x \in X} \right\}$ be an INS. Then, the exponential operational law of the INS *N* with a crisp parameter *μ* is defined as follows:

3 $$ \mu^{N} = \left\{ {\begin{array}{*{20}l} {\left\{ {\left\langle {x,\left[ {\mu^{{1 - T_{N}^{L} (x)}} ,\;\mu^{{1 - T_{N}^{U} (x)}} } \right],\;\left[ {1 - \mu^{{I_{N}^{L} (x)}} ,\;1 - \mu^{{I_{N}^{U} (x)}} } \right],\;\left[ {1 - \mu^{{F_{N}^{L} (x)}} ,\;1 - \mu^{{F_{N}^{U} (x)}} } \right]} \right\rangle |x \in X} \right\},} \hfill & \quad {\mu \in [0,1]} \hfill \\ {\left\{ {\left\langle \begin{aligned} x,\left[ {\left( {1/\mu } \right)^{{1 - T_{N}^{L} (x)}} ,\;\left( {1/\mu } \right)^{{1 - T_{N}^{U} (x)}} } \right],\left[ {1 - \left( {1/\mu } \right)^{{I_{N}^{L} (x)}} ,\;1 - \left( {1/\mu } \right)^{{I_{N}^{U} (x)}} } \right], \hfill \\ \left[ {1 - \left( {1/\mu } \right)^{{F_{N}^{L} (x)}} ,\;1 - \left( {1/\mu } \right)^{{F_{N}^{U} (x)}} } \right] \hfill \\ \end{aligned} \right\rangle |x \in X} \right\},} \hfill &\quad {\mu > 1} \hfill \\ \end{array} .} \right. $$

Obviously, *μ* ^*N*^ is also an INS. Let us discuss the following two cases:

1. If *μ* ∈ [0, 1], the truth-membership, indeterminacy-membership, and falsity-membership functions are $[\mu^{{1 - T_{N}^{L} (x)}} ,\mu^{{1 - T_{N}^{U} (x)}} ]$⊆ [0, 1], $[\mu^{{I_{N}^{L} (x)}} ,\mu^{{I_{N}^{U} (x)}} ]$⊆ [0, 1], and $[\mu^{{F_{N}^{L} (x)}} ,\mu^{{F_{N}^{U} (x)}} ]$⊆ [0, 1] for any *x*∈ *X,* respectively. Thus $\left\{ {\left\langle {x,[\mu^{{1 - T_{N}^{L} (x)}} ,\mu^{{1 - T_{N}^{U} (x)}} ],[1 - \mu^{{I_{N}^{L} (x)}} ,1 - \mu^{{I_{N}^{U} (x)}} ],[1 - \mu^{{F_{N}^{L} (x)}} ,1 - \mu^{{F_{N}^{U} (x)}} ]} \right\rangle |x \in X} \right\}$ is an INS.
2. If *μ* > 1, then there is 0 < 1/*μ* < 1. Obviously, $\left\{ {\left\langle {x,[\left( {1/\mu } \right)^{{1 - T_{N}^{L} (x)}} ,\left( {1/\mu } \right)^{{1 - T_{N}^{U} (x)}} ],[1 - \left( {1/\mu } \right)^{{I_{N}^{L} (x)}} ,1 - \left( {1/\mu } \right)^{{I_{N}^{U} (x)}} ],[1 - \left( {1/\mu } \right)^{{F_{N}^{L} (x)}} ,1 - \left( {1/\mu } \right)^{{F_{N}^{U} (x)}} ]} \right\rangle |x \in X} \right\}$ is also an INS.

Similarly, we can also propose the operational law of an INN.

Let $a = \left\langle {[T_{a}^{L} ,T_{a}^{U} ],[I_{a}^{L} ,I_{a}^{U} ],[F_{a}^{L} ,F_{a}^{U} ]} \right\rangle$ be an INN, which is a basic element in an INS. Then the exponential operational law of the INN *a* with a crisp parameter *μ* is denoted as follows:

4 $$ \mu^{a} = \left\{ {\begin{array}{*{20}l} {\left\langle {x,\left[ {\mu^{{1 - T_{a}^{L} }} ,\;\mu^{{1 - T_{a}^{U} }} } \right],\;\left[ {1 - \mu^{{I_{a}^{L} }} ,\;1 - \mu^{{I_{a}^{U} }} } \right],\;\left[ {1 - \mu^{{F_{a}^{L} }} ,\;1 - \mu^{{F_{a}^{U} }} } \right]} \right\rangle ,} \hfill & {\mu \in [0,1]} \hfill \\ {\left\langle {x,\left[ {\left( {1/\mu } \right)^{{1 - T_{a}^{L} }} ,\;\left( {1/\mu } \right)^{{1 - T_{a}^{U} }} } \right],\;\left[ {1 - \left( {1/\mu } \right)^{{I_{a}^{L} }} ,\;1 - \left( {1/\mu } \right)^{{I_{a}^{U} }} } \right],\;\left[ {1 - \left( {1/\mu } \right)^{{I_{a}^{L} }} ,\;1 - \left( {1/\mu } \right)^{{I_{a}^{U} }} } \right]} \right\rangle ,} \hfill & {\mu > 1} \hfill \\ \end{array} } \right.. $$

It is obvious that *μ* ^*a*^ is also an INN. Let us consider the following example.

#### *Example 1*

Assume that an INN is $a = \langle \left[ {0.5,\;0.6} \right],\;\left[ {0.2,\;0.3} \right],\;\left[ {0.3,\;0.5} \right]\rangle$ and two real numbers are *μ*_1_ = 0.6 and *μ*_2_ = 5 (1/*μ*_2_ = 1/5 = 0.2). Then, by Eq. (4), we can obtain the following results:

$$ \begin{aligned} \mu_{1}^{a} & = 0.6^{\langle [0.5,0.6],[0.2,0.3],[0.3,0.5]\rangle } = \left\langle {\left[ {0.6^{1 - 0.5} ,\;0.6^{1 - 0.6} } \right],\;\left[ {1 - 0.6^{0.2} ,\;1 - 0.6^{0.3} } \right],\;\left[ {1 - 0.6^{0.3} ,\;1 - 0.6^{0.5} } \right]} \right\rangle \\ & = \left\langle {[0.7746, \, 0.8152], \, [0.0971, \, 0.1421], \, [0.1421, \, 0.2254]} \right\rangle , \\ \end{aligned} $$

$$ \begin{aligned} \mu_{2}^{a} & = 5^{\left\langle [0.5,0.6],[0.2,0.3],[0.3,0.5]\right\rangle } = \left\langle {\left[ {0.2^{1 - 0.5} ,\;0.2^{1 - 0.6} } \right],\;\left[ {1 - 0.2^{0.2} ,\;1 - 0.2^{0.3} } \right],\;\left[ {1 - 0.2^{0.3} ,\;1 - 0.2^{0.5} } \right]} \right\rangle \\ & = \left\langle {[0.4472, \, 0.5253], \, [0.2752, \, 0.3830], \, [0.3830, \, 0.5528]} \right\rangle . \\ \end{aligned} $$

It is noted that the bigger the value of *μ*, the smaller the results derived from the exponential operational law.

To compare INNs, we can introduce the following cosine measure function and comparative method for INNs based on the cosine measure function of SNSs (Ye 2015a).

#### **Definition 3**

Let $a = \left\langle {[T_{a}^{L} ,T_{a}^{U} ],[I_{a}^{L} ,I_{a}^{U} ],[F_{a}^{L} ,F_{a}^{U} ]} \right\rangle$ be any INN and the maximum INN be $a^{*} = \left\langle {[1,1],[0,0],[0,0]} \right\rangle$. Then, the cosine measure function based on the Hamming distance between *a* and *a* ^*^ can be defined as

5 $$ C(a) = \cos \left[ {\frac{\pi }{12}\left( {1 - T_{a}^{L} + 1 - T_{a}^{U} + I_{a}^{L} + I_{a}^{U} + F_{a}^{L} + F_{a}^{U} } \right)} \right]\quad {\text{for}}\;C\left( a \right) \in \left[ {0,\;1} \right]. $$

Obviously, the meaning of the cosine measure function *C*(*a*) of any INN *a* can be explained as follows: the closer the INN *a* is to the maximum INN $a^{*} = \left\langle {\left[ {1,\;1} \right],\;\left[ {0,\;0} \right],\;\left[ {0,\;0} \right]} \right\rangle$, the bigger the value of *a*. Hence, we can give a comparative method for INNs according to the cosine measure function *C*(*a*).

#### **Definition 4**

Let $a = \left\langle {[T_{a}^{L} ,T_{a}^{U} ],[I_{a}^{L} ,I_{a}^{U} ],[F_{a}^{L} ,F_{a}^{U} ]} \right\rangle$ and $b = \left\langle {[T_{b}^{L} ,T_{b}^{U} ],[I_{b}^{L} ,I_{b}^{U} ],[F_{b}^{L} ,F_{b}^{U} ]} \right\rangle$ be two INNs, then the comparative method based on the cosine measure function *C*(*a*) can be defined as follows:

1. If *C*(*a*) > *C*(*b*), then *a* ≻ *b*,
2. If *C*(*a*) = *C*(*b*), then *a* = *b*.

In the following, we only discuss some basic properties of the exponential operational laws of INNs when *μ* ∈ [0, 1] because the properties of *μ* ^*a*^ for *μ* > 1 are similar to the case of *μ* ∈ [0, 1].

#### **Theorem 1**

*Let* $a = \left\langle {[T_{a}^{L} ,T_{a}^{U} ],[I_{a}^{L} ,I_{a}^{U} ],[F_{a}^{L} ,F_{a}^{U} ]} \right\rangle$ *and* $b = \left\langle {[T_{b}^{L} ,T_{b}^{U} ],[I_{b}^{L} ,I_{b}^{U} ],[F_{b}^{L} ,F_{b}^{U} ]} \right\rangle$ *be two INNs and μ ∈* [0, 1] *be a real number, then there are the following commutative laws:*

1. $$ \mu^{a} \oplus \mu^{b} = \mu^{b} \oplus \mu^{a} $$;
2. $$ \mu^{a} \otimes \mu^{b} = \mu^{b} \otimes \mu^{a} $$.

*Obviously, the commutative laws are true. Hence, their proofs are omitted here.*

#### **Theorem 2**

*Let* $a = \left\langle {[T_{a}^{L} ,T_{a}^{U} ],[I_{a}^{L} ,I_{a}^{U} ],[F_{a}^{L} ,F_{a}^{U} ]} \right\rangle$, $b = \left\langle {[T_{b}^{L} ,T_{b}^{U} ],[I_{b}^{L} ,I_{b}^{U} ],[F_{b}^{L} ,F_{b}^{U} ]} \right\rangle$ *, and* $c = \left\langle {[T_{c}^{L} ,T_{c}^{U} ],[I_{c}^{L} ,I_{c}^{U} ],[F_{c}^{L} ,F_{c}^{U} ]} \right\rangle$ *be three INNs and μ ∈* [0, 1] *be a real number, then there are the following associative laws:*

1. $$ (\mu^{a} \oplus \mu^{b} ) \oplus \mu^{c} = \mu^{a} \oplus (\mu^{b} \oplus \mu^{c} ) $$;
2. $$ (\mu^{a} \otimes \mu^{b} ) \otimes \mu^{c} = \mu^{a} \otimes (\mu^{b} \otimes \mu^{c} ) $$.

*It is obvious that the associative laws are also true. Hence, their proofs are omitted.*

#### **Theorem 3**

*Let* $a = \left\langle {[T_{a}^{L} ,T_{a}^{U} ],[I_{a}^{L} ,I_{a}^{U} ],[F_{a}^{L} ,F_{a}^{U} ]} \right\rangle$ *be an INN. When μ*_1_ ≥ *μ*_2_, *we can obtain* (*μ*_1_)^*a*^ ≥ (*μ*_2_)^*a*^ *for μ*_1_, *μ*_2_ ∈ [0, 1] *and* (*μ*_1_)^*a*^ ≤ (*μ*_2_)^*a*^ *for μ*_1_, *μ*_2_ > 1.

#### *Proof*

If *μ*_1_ ≥ *μ*_2_ and *μ*_1_, *μ*_2_ ∈ [0, 1], based on the exponential operational law of INN, we have the following operational results:

$$ (\mu_{1} )^{a} = \left\langle {\left[ {(\mu_{1} )^{{1 - T_{a}^{L} }} ,\;(\mu_{1} )^{{1 - T_{a}^{U} }} } \right],\;\left[ {1 - (\mu_{1} )^{{I_{a}^{L} }} ,\;1 - (\mu_{1} )^{{I_{a}^{U} }} } \right],\;\left[ {1 - (\mu_{1} )^{{I_{a}^{L} }} ,\;1 - (\mu_{1} )^{{I_{a}^{U} }} } \right]} \right\rangle , $$

$$ (\mu_{2} )^{a} = \left\langle {\left[ {(\mu_{2} )^{{1 - T_{a}^{L} }} ,\;(\mu_{2} )^{{1 - T_{a}^{U} }} } \right],\;\left[ {1 - (\mu_{2} )^{{I_{a}^{L} }} ,\;1 - (\mu_{2} )^{{I_{a}^{U} }} } \right],\;\left[ {1 - (\mu_{2} )^{{F_{a}^{L} }} ,\;1 - (\mu_{2} )^{{F_{a}^{U} }} } \right]} \right\rangle . $$

Since $(\mu_{1} )^{{1 - T_{a}^{L} }} \ge (\mu_{2} )^{{1 - T_{a}^{L} }}$, $(\mu_{1} )^{{1 - T_{a}^{U} }} \ge (\mu_{2} )^{{1 - T_{a}^{U} }}$ $1 - (\mu_{1} )^{{I_{a}^{L} }} \le 1 - (\mu_{2} )^{{I_{a}^{L} }}$, $1 - (\mu_{1} )^{{I_{a}^{U} }} \le 1 - (\mu_{2} )^{{I_{a}^{U} }}$, $1 - (\mu_{1} )^{{F_{a}^{L} }} \le 1 - (\mu_{2} )^{{F_{a}^{L} }}$, and $1 - (\mu_{1} )^{{F_{a}^{U} }} \le 1 - (\mu_{2} )^{{F_{a}^{U} }}$ for *μ*_1_ ≥ *μ*_2_, by Eq. (5) there is the following cosine measure relation:

$$ \begin{aligned} C((\mu_{1} )^{a} ) & = \cos \left\{ {\left[ {1 - (\mu_{1} )^{{1 - T_{a}^{L} }} + \left( {1 - (\mu_{1} )^{{I_{a}^{L} }} } \right) + \left( {1 - (\mu_{1} )^{{F_{a}^{L} }} } \right) + 1 - (\mu_{1} )^{{1 - T_{a}^{U} }} + \left( {1 - (\mu_{1} )^{{I_{a}^{U} }} } \right) + \left( {1 - (\mu_{1} )^{{F_{a}^{U} }} } \right)} \right]\pi /12} \right\} \\ \ge C((\mu_{2} )^{a} ) & = \cos \left\{ {\left[ {1 - (\mu_{2} )^{{1 - T_{a}^{L} }} + \left( {1 - (\mu_{2} )^{{I_{a}^{L} }} } \right) + \left( {1 - (\mu_{2} )^{{F_{a}^{L} }} } \right) + 1 - (\mu_{2} )^{{1 - T_{a}^{U} }} + \left( {1 - (\mu_{2} )^{{I_{a}^{U} }} } \right) + \left( {1 - (\mu_{2} )^{{F_{a}^{U} }} } \right)} \right]\pi /12} \right\}. \\ \end{aligned} $$

Obviously, there is (*μ*_1_)^*a*^ ≥ (*μ*_2_)^*a*^ for *μ*_1_ ≥ *μ*_2_ and *μ*_1_, *μ*_2_ ∈ [0, 1].

When *μ*_1_, *μ*_2_ > 1 and *μ*_1_ ≥ *μ*_2_, we can know 0 < 1/*μ*_1_ ≤ 1/*μ*_2_ ≤ 1. As discussed above, we can also obtain (*μ*_1_)^*a*^ ≤ (*μ*_2_)^*a*^.

This completes the proofs. □

Taking some values of *μ*, we can reveals some special values of *μ* ^*a*^:

1. If *μ* = 1, then $\mu^{a} = \left\langle {[\mu^{{1 - T_{a}^{L} }} ,\mu^{{1 - T_{a}^{U} }} ],[1 - \mu^{{I_{a}^{L} }} ,1 - \mu^{{I_{a}^{U} }} ],[1 - \mu^{{I_{a}^{L} }} ,1 - \mu^{{I_{a}^{U} }} ]} \right\rangle = \left\langle {[1,1],[0,0],[0,0]} \right\rangle$ for each INN *a*;
2. If $a = \left\langle { \left[ {1,\;1} \right],\;\left[ {0,\;0} \right],\;\left[ {0,\;0} \right]} \right\rangle$ , then $\mu^{a} = \left\langle {[\mu^{{1 - T_{a}^{L} }} ,\mu^{{1 - T_{a}^{U} }} ],[1 - \mu^{{I_{a}^{L} }} ,1 - \mu^{{I_{a}^{U} }} ],[1 - \mu^{{I_{a}^{L} }} ,1 - \mu^{{I_{a}^{U} }} ]} \right\rangle = \left\langle { \left[ {1,\;1} \right],\;\left[ {0,\;0} \right],\;\left[ {0,\;0} \right]} \right\rangle$ for each value of *μ*;
3. If $a = \left\langle { \left[ {0,\;0} \right],\;\left[ {1,\;1} \right],\;\left[ {1,\;1} \right]} \right\rangle$, then $\mu^{s} = \left\langle {[\mu^{{1 - T_{a}^{L} }} ,\mu^{{1 - T_{a}^{U} }} ],[1 - \mu^{{I_{a}^{L} }} ,1 - \mu^{{I_{a}^{U} }} ],[1 - \mu^{{F_{a}^{L} }} ,1 - \mu^{{F_{a}^{U} }} ]} \right\rangle$ $= \left\langle {[\mu ,\mu ],[1 - \mu ,1 - \mu ],[1 - \mu ,1 - \mu ]} \right\rangle$ for each value of *μ*.

### Exponential operations of INNs with interval-valued parameters

Firstly, we define a DINN to give the exponential operational law of an INN with interval-valued parameters.

#### **Definition 5**

Let $a = \left\langle {[T_{a}^{L} ,T_{a}^{U} ],[I_{a}^{L} ,I_{a}^{U} ],[F_{a}^{L} ,F_{a}^{U} ]} \right\rangle$ and $b = \left\langle {[T_{b}^{L} ,T_{b}^{U} ],[I_{b}^{L} ,I_{b}^{U} ],[F_{b}^{L} ,F_{b}^{U} ]} \right\rangle$ be two INNs. If $[T_{a}^{L} ,T_{a}^{U} ] \le [T_{b}^{L} ,T_{b}^{U} ]$, $[I_{a}^{L} ,I_{a}^{U} ] \ge [I_{b}^{L} ,I_{b}^{U} ]$, and $[F_{a}^{L} ,F_{a}^{U} ] \ge [F_{b}^{L} ,F_{b}^{U} ]$, there is *a* ≤ *b*. Then we call $\tilde{d}$ = [*a*, *b*] a DINN.

If the exponent is represented by an INN and the base is used by an interval number, then we can give the following exponential operational law of an INN.

#### **Definition 6**

Let $a = \left\langle {[T_{a}^{L} ,T_{a}^{U} ],[I_{a}^{L} ,I_{a}^{U} ],[F_{a}^{L} ,F_{a}^{U} ]} \right\rangle$ be an INN and $\tilde{\mu }$ = [*μ* ^L^, *μ* ^U^] be an interval number, then the exponential operational law of the INN *a* is defined as

6 $$ \tilde{\mu }^{a} = \left\{ {\begin{array}{*{20}l} {\left\{ \begin{aligned} \left\langle {\left[ {\left( {\mu^{L} } \right)^{{1 - T_{a}^{L} }} ,\;\left( {\mu^{L} } \right)^{{1 - T_{a}^{U} }} } \right],\;\left[ {1 - \left( {\mu^{L} } \right)^{{I_{a}^{L} }} ,\;1 - \left( {\mu^{L} } \right)^{{I_{a}^{U} }} } \right],\;\left[ {1 - \left( {\mu^{L} } \right)^{{F_{a}^{L} }} ,\;1 - \left( {\mu^{L} } \right)^{{F_{a}^{U} }} } \right]} \right\rangle , \hfill \\ \left\langle {\left[ {\left( {\mu^{U} } \right)^{{1 - T_{a}^{L} }} ,\;\left( {\mu^{U} } \right)^{{1 - T_{a}^{U} }} } \right],\;\left[ {1 - \left( {\mu^{U} } \right)^{{I_{a}^{L} }} ,\;1 - \left( {\mu^{U} } \right)^{{I_{a}^{U} }} } \right],\;\left[ {1 - \left( {\mu^{U} } \right)^{{F_{a}^{L} }} ,\;1 - \left( {\mu^{U} } \right)^{{F_{a}^{U} }} } \right]} \right\rangle \hfill \\ \end{aligned} \right\},} \hfill & {if\;0 \le \mu^{L} \le \mu^{U} \le 1} \hfill \\ {\left\{ \begin{aligned} \left\langle {\left[ {\left( {1 /\mu^{U} } \right)^{{1 - T_{a}^{L} }} ,\;\left( {1 /\mu^{U} } \right)^{{1 - T_{a}^{U} }} } \right],\;\left[ {1 - \left( {1 /\mu^{U} } \right)^{{I_{a}^{L} }} ,\;1 - \left( {1 /\mu^{U} } \right)^{{I_{a}^{U} }} } \right],\;\left[ {1 - \left( {1 /\mu^{U} } \right)^{{F_{a}^{L} }} ,\;1 - \left( {1 /\mu^{U} } \right)^{{F_{a}^{U} }} } \right]} \right\rangle , \hfill \\ \left\langle {\left[ {\left( {1/\mu^{L} } \right)^{{1 - T_{a}^{L} }} ,\left( {1 /\mu^{L} } \right)^{{1 - T_{a}^{U} }} } \right],\;\left[ {1 - \left( {1 /\mu^{L} } \right)^{{I_{a}^{L} }} ,\;1 - \left( {1 /\mu^{L} } \right)^{{I_{a}^{U} }} } \right],\;\left[ {1 - \left( {1 /\mu^{L} } \right)^{{F_{a}^{L} }} ,\;1 - \left( {1 /\mu^{L} } \right)^{{F_{a}^{U} }} } \right]} \right\rangle \hfill \\ \end{aligned} \right\},} \hfill & {if\;1 < \mu^{L} \le \mu^{U} } \hfill \\ \end{array} .} \right. $$

Obviously, the value of $\tilde{\mu }^{a}$ is a DINN since it is similar to the exponential operational laws of two INNs.

#### *Example 2*

Let $a = \left\langle { \left[ {0.5,\;0.6} \right],\;\left[ {0.2,\;0.3} \right],\;\left[ {0.3,\;0.5} \right]} \right\rangle$ be an INN and two interval numbers are $\tilde{\mu }_{1}^{a}$ = [0.4, 0.6] and $\tilde{\mu }_{2}^{a}$ = [4, 6]. Then, by Eq. (6), we can obtain the following results:

$$ \begin{aligned} \tilde{\mu }_{1}^{a} & = [0.4,\;0.6]^{\langle [0.5,0.6],[0.2,0.3],[0.3,0.5]\rangle } \\ & = \left\{ {\begin{array}{*{20}l} {\left\langle {\left[ {0.4^{1 - 0.5} ,\;0.4^{1 - 0.6} } \right],\;\left[ {1 - 0.4^{0.2} ,\;1 - 0.4^{0.3} } \right],\;\left[ {1 - 0.4^{0.3} ,\;1 - 0.4^{0.5} } \right]} \right\rangle ,} \hfill \\ {\left\langle {\left[ {0.6^{1 - 0.5} ,\;0.6^{1 - 0.6} } \right],\;\left[ {1 - 0.6^{0.2} ,\;1 - 0.6^{0.3} } \right],\;\left[ {1 - 0.6^{0.3} ,\;1 - 0.6^{0.5} } \right]} \right\rangle } \hfill \\ \end{array} } \right\} \\ & = \left\{ {\begin{array}{*{20}l} {\left\langle { 0. 6 3 2 5 ,\; 0. 6 9 3 1 ] ,\; [ 0. 1 6 7 4 ,\; 0. 2 4 0 3 ] ,\; [ 0. 2 4 0 3 ,\; 0. 3 6 7 5 ]} \right\rangle ,} \hfill \\ {\left\langle { [ 0. 7 7 4 6 ,\; 0. 8 1 5 2 ] ,\; [ 0. 0 9 7 1 ,\; 0. 1 4 2 1 ] ,\; [ 0. 1 4 2 1 ,\; 0. 2 2 5 4 ]} \right\rangle } \hfill \\ \end{array} } \right\}, \\ \end{aligned} $$

$$ \begin{aligned} \tilde{\mu }_{2}^{a} &= [4,6]^{ < [0.5,0.6],[0.2,0.3],[0.3,0.5] > } \hfill \\ \quad \; &= \left\{ \begin{aligned} \left\langle {[(1/6)^{1 - 0.5} ,(1/6)^{1 - 0.6} ],[1 - (1/6)^{0.2} ,1 - (1/6)^{0.3} ],[1 - (1/6)^{0.3} ,1 - (1/6)^{0.5} ]} \right\rangle , \hfill \\ \left\langle {[(1/4)^{1 - 0.5} ,(1/4)^{1 - 0.6} ],[1 - (1/4)^{0.2} ,1 - (1/4)^{0.3} ],[1 - (1/4)^{0.3} ,1 - (1/4)^{0.5} ]} \right\rangle \hfill \\ \end{aligned} \right\} \hfill \\ \quad \; &= \left\{ \begin{aligned} \left\langle { [ 0. 4 0 8 2 , { 0} . 4 8 8 4 ] , { [0} . 3 0 1 2 , { 0} . 4 1 5 8 ] , { [0} . 4 1 5 8 , { 0} . 5 9 1 8 ]} \right\rangle , \hfill \\ \left\langle { [ 0. 5 0 0 0 , { 0} . 5 7 4 3 ] , { [0} . 2 4 2 1 , { 0} . 3 4 0 2 ] , { [0} . 3 4 0 2 , { 0} . 5 0 0 0 ]} \right\rangle \hfill \\ \end{aligned} \right\}. \hfill \\ \end{aligned} $$

Then, we need to define some basic operations, such as addition and multiplication, for DINNs.

#### **Definition 7**

Let $\tilde{d}_{j} = [a_{j}^{L} ,a_{j}^{U} ]$ (*j* = 1, 2) be DINNs and μ be a real number. Then we can define the following operations:

1. $$ \tilde{d}_{1} \oplus \tilde{d}_{2} = [a_{1}^{L} ,a_{1}^{U} ] \oplus [a_{2}^{L} ,a_{2}^{U} ] = [a_{1}^{L} \oplus a_{2}^{L} ,a_{1}^{U} \oplus a_{2}^{U} ] $$;
2. $$ \tilde{d}_{1} \otimes \tilde{d}_{2} = [a_{1}^{L} ,a_{1}^{U} ] \otimes [a_{2}^{L} ,a_{2}^{U} ] = [a_{1}^{L} \otimes a_{2}^{L} ,a_{1}^{U} \otimes a_{2}^{U} ] $$;
3. $$ \mu \tilde{d}_{1} = \mu [a_{1}^{L} ,a_{1}^{U} ] = [\mu a_{1}^{L} ,\mu a_{1}^{U} ] $$;
4. $$ (\tilde{d}_{1} )^{\mu } = [a_{1}^{L} ,a_{1}^{U} ]^{\mu } = [(a_{1}^{L} )^{\mu } ,(a_{1}^{U} )^{\mu } ] $$.

In the above operations, we firstly use the interval operational laws and the rest of the operations are calculated between INNs or real numbers. Hence they not only ensure the rationality of interval operations, but also conform to the operational laws of INNs.

Similarly, the exponential operational law of INN with interval-valued parameters also satisfies some properties.

#### **Theorem 4**

*Let* $a = \left\langle {[T_{a}^{L} ,T_{a}^{U} ],[I_{a}^{L} ,I_{a}^{U} ],[F_{a}^{L} ,F_{a}^{U} ]} \right\rangle$ *and* $b = \left\langle {[T_{b}^{L} ,T_{b}^{U} ],[I_{b}^{L} ,I_{b}^{U} ],[F_{b}^{L} ,F_{b}^{U} ]} \right\rangle$ *be two INNs and* $\tilde{\mu }_{j} = [\mu_{j}^{L} ,\mu_{j}^{U} ]$(*j* = 1, 2) *be interval numbers for* $0 \le \mu_{j}^{L} \le \mu_{j}^{U} \le 1$. *Then there are the following commutative laws:*

1. $$ (\tilde{\mu }_{1} )^{a} \oplus (\tilde{\mu }_{2} )^{b} = (\tilde{\mu }_{2} )^{b} \oplus (\tilde{\mu }_{1} )^{a} $$;
2. $$ (\tilde{\mu }_{1} )^{a} \otimes (\tilde{\mu }_{2} )^{b} = (\tilde{\mu }_{2} )^{b} \otimes (\tilde{\mu }_{1} )^{a} $$.

#### **Theorem 5**

*Let* $a = \left\langle {[T_{a}^{L} ,T_{a}^{U} ],[I_{a}^{L} ,I_{a}^{U} ],[F_{a}^{L} ,F_{a}^{U} ]} \right\rangle$, $b = \left\langle {[T_{b}^{L} ,T_{b}^{U} ],[I_{b}^{L} ,I_{b}^{U} ],[F_{b}^{L} ,F_{b}^{U} ]} \right\rangle$ *, and* $c = \left\langle {[T_{c}^{L} ,T_{c}^{U} ],[I_{c}^{L} ,I_{c}^{U} ],[F_{c}^{L} ,F_{c}^{U} ]} \right\rangle$ *be three INNs and* $\tilde{\mu }_{j} = [\mu_{j}^{L} ,\mu_{j}^{U} ]$(*j* = 1, 2, 3) *be interval numbers for* $0 \le \mu_{j}^{L} \le \mu_{j}^{U} \le 1$ *. Then there are the following associative laws:*

$$ [(\tilde{\mu }_{1} )^{a} \oplus (\tilde{\mu }_{2} )^{b} ] \oplus (\tilde{\mu }_{3} )^{c} = (\tilde{\mu }_{1} )^{a} \oplus [(\tilde{\mu }_{2} )^{b} \oplus (\tilde{\mu }_{3} )^{c} ] $$;

$$ [(\tilde{\mu }_{1} )^{a} \otimes (\tilde{\mu }_{2} )^{b} ] \otimes (\tilde{\mu }_{3} )^{c} = (\tilde{\mu }_{1} )^{a} \otimes [(\tilde{\mu }_{2} )^{b} \otimes (\tilde{\mu }_{3} )^{c} ] $$.

*Obviously, the above two theorems are very straightforward. Hence, their proofs are omitted.*

*Based on the above cosine measure function and corresponding comparative method for INNs, we can also propose the following cosine measure function and corresponding comparative method for DINNs.*

#### **Definition 8**

Let $\tilde{d} = \left\{ {\left\langle {[T_{{a_{{}}^{L} }}^{L} ,T_{{a_{{}}^{L} }}^{U} ],[I_{{a_{{}}^{L} }}^{L} ,I_{{a_{{}}^{L} }}^{U} ],[F_{{a_{{}}^{L} }}^{L} ,F_{{a_{{}}^{L} }}^{U} ]} \right\rangle ,\left\langle {[T_{{a_{{}}^{U} }}^{L} ,T_{{a_{{}}^{U} }}^{U} ],[I_{{a_{{}}^{U} }}^{L} ,I_{{a_{{}}^{U} }}^{U} ],[F_{{a_{{}}^{U} }}^{L} ,F_{{a_{{}}^{U} }}^{U} ]} \right\rangle } \right\}$ be a DINN and $\tilde{d}^{*} = \left\{ {\left\langle {[1,1],[0,0],[0,0]} \right\rangle ,\left\langle {[1,1],[0,0],[0,0]} \right\rangle } \right\}$ be the maximum DINN. The cosine measure function based on the Hamming distance between $\tilde{d}$ and $\tilde{d}^{*}$ can be defined as

7 $$ C(\tilde{d}) = \cos \left\{ {\begin{array}{*{20}c} {\frac{\pi }{24}(1 - T_{{a^{L} }}^{L} + 1 - T_{{a^{L} }}^{U} + I_{{a^{L} }}^{L} + I_{{a^{L} }}^{U} + F_{{a^{L} }}^{L} + F_{{a^{L} }}^{U} } \\ { + 1 - T_{{a^{U} }}^{L} + 1 - T_{{a^{U} }}^{U} + I_{{a^{U} }}^{L} + I_{{a^{U} }}^{U} + F_{{a^{U} }}^{L} + F_{{a^{U} }}^{U} )} \\ \end{array} } \right\}\quad {\text{for}}\;C(\tilde{d}) \in [0,1]. $$

Similarly, the meaning of the cosine measure function $C(\tilde{d})$ of the DINN $\tilde{d}$ can be explained as follows: the closer the DINN $\tilde{d}$ is to the maximum DINN $\tilde{d}^{*} = \left\{ {\left\langle { \left[ {1,\;1} \right],\;\left[ {0,\;0} \right],\;\left[ {0,\;0} \right]} \right\rangle ,\;\left\langle {\left[ {1,\;1} \right],\;\left[ {0,\;0} \right],\;\left[ {0,\;0} \right]} \right\rangle } \right\}$, the bigger the value of $\tilde{d}$. Hence, we can give a comparative method for DINNs.

#### **Definition 9**

Let $\tilde{d}_{1} = \left\{ {\left\langle {[T_{{a_{1}^{L} }}^{L} ,T_{{a_{1}^{L} }}^{U} ],[I_{{a_{1}^{L} }}^{L} ,I_{{a_{1}^{L} }}^{U} ],[F_{{a_{1}^{L} }}^{L} ,F_{{a_{1}^{L} }}^{U} ]} \right\rangle ,\left\langle {[T_{{a_{1}^{U} }}^{L} ,T_{{a_{1}^{U} }}^{U} ],[I_{{a_{1}^{U} }}^{L} ,I_{{a_{1}^{U} }}^{U} ],[F_{{a_{1}^{U} }}^{L} ,F_{{a_{1}^{U} }}^{U} ]} \right\rangle } \right\}$ and $\tilde{d}_{2} = \left\{ {\left\langle {[T_{{a_{2}^{L} }}^{L} ,T_{{a_{2}^{L} }}^{U} ],[I_{{a_{2}^{L} }}^{L} ,I_{{a_{2}^{L} }}^{U} ],[F_{{a_{2}^{L} }}^{L} ,F_{{a_{2}^{L} }}^{U} ]} \right\rangle ,\left\langle {[T_{{a_{2}^{U} }}^{L} ,T_{{a_{2}^{U} }}^{U} ],[I_{{a_{2}^{U} }}^{L} ,I_{{a_{2}^{U} }}^{U} ],[F_{{a_{2}^{U} }}^{L} ,F_{{a_{2}^{U} }}^{U} ]} \right\rangle } \right\}$ be two DINNs. Then the comparative method based on the cosine measure functions for $\tilde{d}_{1}$ and $\tilde{d}_{2}$ can be defined as follows:

1. If $$ C(\tilde{d}_{1} ) > C(\tilde{d}_{2} ) $$, then $$ \tilde{d}_{1}\succ \tilde{d}_{2}$$;
2. If $$ C(\tilde{d}_{1} ) = C(\tilde{d}_{2} ) $$, then $$ \tilde{d}_{1} $$ = $$ \tilde{d}_{2} $$.

## Exponential aggregation operators of INNs

According to the exponential operational laws of Eqs. (4) and (6), this section proposes the INWEA and DINWEA operators, where the bases are a collection of real numbers or interval numbers and the exponents are a collection of INNs.

### **Definition 10**

Let $a_{j} = \left\langle {[T_{{a_{j} }}^{L} ,T_{{a_{j} }}^{U} ],[I_{{a_{j} }}^{L} ,I_{{a_{j} }}^{U} ],[F_{{a_{j} }}^{L} ,F_{{a_{j} }}^{U} ]} \right\rangle$ for *j* = 1, 2, …, *n* be a collection of INNs and *μ*_*j*_ for *j* = 1, 2, …, *n* be a collection of real numbers, and let a mapping INWEA: Ω^*n*^ → Ω. Then, the INWEA operator is defined as

8 $$ INWEA(a_{1} ,a_{2} , \ldots ,a_{n} ) = \prod\limits_{j = 1}^{n} {(\mu_{j} )^{{a_{j} }} } , $$

where *a*_*j*_ (*j* = 1, 2, …, *n*) is the exponential weight of *μ*_*j*_ (*j* = 1, 2, …, *n*).

### **Theorem 6**

*Let* $a_{j} = \left\langle {[T_{{a_{j} }}^{L} ,T_{{a_{j} }}^{U} ],[I_{{a_{j} }}^{L} ,I_{{a_{j} }}^{U} ],[F_{{a_{j} }}^{L} ,F_{{a_{j} }}^{U} ]} \right\rangle$ *for j* = 1, 2, …, *n be a collection of INNs and μ*_*j*_ *for j* = 1,2, …, *n be a collection of real numbers, then the aggregated value of the INWEA operator is also an INN, where*

9 $$ INWEA(a_{1} ,a_{2} , \ldots ,a_{n} ) = \left\{ {\begin{array}{*{20}c} {\left\langle {\begin{array}{*{20}l} {\left[ {\prod\limits_{j = 1}^{n} {(\mu_{j} )^{{1 - T_{{a_{j} }}^{L} }} } ,\;\prod\limits_{j = 1}^{n} {(\mu_{j} )^{{1 - T_{{a_{j} }}^{U} }} } } \right],} \hfill \\ {\left[ {1 - \prod\limits_{j = 1}^{n} {(\mu_{j} )^{{I_{{a_{j} }}^{L} }} } ,\;1 - \prod\limits_{j = 1}^{n} {(\mu_{j} )^{{I_{{a_{j} }}^{U} }} } } \right],} \hfill \\ {\left[ {1 - \prod\limits_{j = 1}^{n} {(\mu_{j} )^{{F_{{a_{j} }}^{L} }} } ,\;1 - \prod\limits_{j = 1}^{n} {(\mu_{j} )^{{F_{{a_{j} }}^{U} }} } } \right]} \hfill \\ \end{array} } \right\rangle ,} & {if\;\mu \in [0,1]} \\ {\left\langle {\begin{array}{*{20}l} {\left[ {\prod\limits_{j = 1}^{n} {(1 /\mu_{j} )^{{1 - T_{{a_{j} }}^{L} }} } ,\;\prod\limits_{j = 1}^{n} {(1 /\mu_{j} )^{{1 - T_{{a_{j} }}^{U} }} } } \right],} \hfill \\ {\left[ {1 - \prod\limits_{j = 1}^{n} {(1 /\mu_{j} )^{{I_{{a_{j} }}^{L} }} } ,\;1 - \prod\limits_{j = 1}^{n} {(1 /\mu_{j} )^{{I_{{a_{j} }}^{U} }} } } \right],} \hfill \\ {\left[ {1 - \prod\limits_{j = 1}^{n} {(1 /\mu_{j} )^{{F_{{a_{j} }}^{L} }} } ,\;1 - \prod\limits_{j = 1}^{n} {(1 /\mu_{j} )^{{F_{{a_{j} }}^{U} }} } } \right]} \hfill \\ \end{array} } \right\rangle ,} & {if\;\mu > 1} \\ \end{array} } \right. $$

*and a*_*j*_ (*j* = 1, 2, …, *n*) *is the exponential weight of μ*_*j*_ (*j* = 1, 2, …, *n*).

### *Proof*

By using mathematical induction, we can prove Eq. (9) if *μ*_*j*_ ∈ [0, 1] for *j* = 1, 2, …, *n*.

1. If *n* = 2, we can obtain

10 $$ \begin{aligned} INWEA(a_{1} ,a_{2} ) & = (\mu_{1} )^{{a_{1} }} \otimes (\mu_{2} )^{{a_{1} }} \\ & = \left\langle {\begin{array}{*{20}l} {\left[ {(\mu_{1} )^{{1 - T_{{a_{1} }}^{L} }} (\mu_{2} )^{{1 - T_{{a_{2} }}^{L} }} ,(\mu_{1} )^{{1 - T_{{a_{1} }}^{U} }} (\mu_{2} )^{{1 - T_{{a_{2} }}^{U} }} } \right],} \hfill \\ {[1 - (\mu_{1} )^{{I_{{a_{1} }}^{L} }} + 1 - (\mu_{2} )^{{I_{{a_{2} }}^{L} }} - \left( {1 - (\mu_{1} )^{{I_{{a_{1} }}^{L} }} } \right)\left( {1 - (\mu_{2} )^{{I_{{a_{2} }}^{L} }} } \right),} \hfill \\ {1 - (\mu_{1} )^{{I_{{a_{1} }}^{U} }} + 1 - (\mu_{2} )^{{I_{{a_{2} }}^{U} }} - \left( {1 - (\mu_{1} )^{{I_{{a_{1} }}^{U} }} } \right)\left( {1 - (\mu_{2} )^{{I_{{a_{2} }}^{U} }} } \right)],} \hfill \\ {[1 - (\mu_{1} )^{{F_{{a_{1} }}^{L} }} + 1 - (\mu_{2} )^{{F_{{a_{2} }}^{L} }} - \left( {1 - (\mu_{1} )^{{F_{{a_{1} }}^{L} }} } \right)\left( {1 - (\mu_{2} )^{{F_{{a_{2} }}^{L} }} } \right),} \hfill \\ {1 - (\mu_{1} )^{{F_{{a_{1} }}^{U} }} + 1 - (\mu_{2} )^{{F_{{a_{2} }}^{U} }} - \left( {1 - (\mu_{1} )^{{F_{{a_{1} }}^{U} }} } \right)\left( {1 - (\mu_{2} )^{{F_{{a_{2} }}^{U} }} } \right)]} \hfill \\ \end{array} } \right\rangle \\ & = \left\langle {\left[ {\prod\limits_{j = 1}^{2} {(\mu_{j} )^{{1 - T_{{a_{j} }}^{L} }} } ,\;\prod\limits_{j = 1}^{2} {(\mu_{j} )^{{1 - T_{{a_{j} }}^{U} }} } } \right],\;\left[ {1 - \prod\limits_{j = 1}^{2} {(\mu_{j} )^{{I_{{a_{j} }}^{L} }} } ,\;1 - \prod\limits_{j = 1}^{2} {(\mu_{j} )^{{I_{{a_{j} }}^{U} }} } } \right],\;\left[ {1 - \prod\limits_{j = 1}^{2} {(\mu_{j} )^{{F_{{a_{j} }}^{L} }} } ,\;1 - \prod\limits_{j = 1}^{2} {(\mu_{j} )^{{F_{{a_{j} }}^{U} }} } } \right]} \right\rangle . \\ \end{aligned} $$

2. If *n* = *k*, by Eq. (9) there is the following formula:

11 $$ INWEA(a_{1} ,a_{2} , \ldots ,a_{k} ) = \left\langle {\begin{array}{*{20}c} {\left[ {\prod\limits_{j = 1}^{k} {(\mu_{j} )^{{1 - T_{{a_{j} }}^{L} }} } ,\;\prod\limits_{j = 1}^{k} {(\mu_{j} )^{{1 - T_{{a_{j} }}^{U} }} } } \right],} \\ {\left[ {1 - \prod\limits_{j = 1}^{k} {(\mu_{j} )^{{I_{{a_{j} }}^{L} }} } ,\;1 - \prod\limits_{j = 1}^{k} {(\mu_{j} )^{{I_{{a_{j} }}^{U} }} } } \right],} \\ {\left[ {1 - \prod\limits_{j = 1}^{k} {(\mu_{j} )^{{F_{{a_{j} }}^{L} }} } ,\;1 - \prod\limits_{j = 1}^{k} {(\mu_{j} )^{{F_{{a_{j} }}^{U} }} } } \right]} \\ \end{array} } \right\rangle . $$

3. If *n* = *k* + 1, by the operational law of Eq. (4) and combining Eqs. (10) and (11), we have

$$ \begin{aligned} INWEA(a_{1} ,a_{2} , \ldots ,a_{k} ,a_{k + 1} ) & = \left\langle {\begin{array}{*{20}l} {\left[ {\prod\limits_{j = 1}^{k} {(\mu_{j} )^{{1 - T_{{a_{j} }}^{L} }} } ,\;\prod\limits_{j = 1}^{k} {(\mu_{j} )^{{1 - T_{{a_{j} }}^{U} }} } } \right],} \hfill \\ {\left[ {1 - \prod\limits_{j = 1}^{k} {(\mu_{j} )^{{I_{{a_{j} }}^{L} }} } ,\;1 - \prod\limits_{j = 1}^{k} {(\mu_{j} )^{{I_{{a_{j} }}^{U} }} } } \right],} \hfill \\ {\left[ {1 - \prod\limits_{j = 1}^{k} {(\mu_{j} )^{{F_{{a_{j} }}^{L} }} } ,\;1 - \prod\limits_{j = 1}^{k} {(\mu_{j} )^{{F_{{a_{j} }}^{U} }} } } \right]} \hfill \\ \end{array} } \right\rangle \otimes (\mu_{k + 1} )^{{a_{{_{k + 1} }} }} \\ & = \left\langle {\left[ {\prod\limits_{j = 1}^{k + 1} {(\mu_{j} )^{{1 - T_{{a_{j} }}^{L} }} } ,\;\prod\limits_{j = 1}^{k + 1} {(\mu_{j} )^{{1 - T_{{a_{j} }}^{U} }} } } \right],\;\left[ {1 - \prod\limits_{j = 1}^{k + 1} {(\mu_{j} )^{{I_{{a_{j} }}^{L} }} } ,\;1 - \prod\limits_{j = 1}^{k + 1} {(\mu_{j} )^{{I_{{a_{j} }}^{U} }} } } \right],\;\left[ {1 - \prod\limits_{j = 1}^{k + 1} {(\mu_{j} )^{{F_{{a_{j} }}^{L} }} } ,\;1 - \prod\limits_{j = 1}^{k + 1} {(\mu_{j} )^{{F_{{a_{j} }}^{U} }} } } \right]} \right\rangle . \\ \end{aligned} $$

From the above results, we can obtain that Eq. (9) holds for any *n* for *μ*_*j*_ ∈ [0, 1].

If *μ*_*j*_ > 1 and 0 < 1/*μ*_*j*_ < 1 for *j* = 1, 2, …, *n*, by the above similar proof we can also obtain the following aggregation operator:

$$ INWEA(a_{1} ,a_{2} , \ldots ,a_{n} ) = \left\langle {\begin{array}{*{20}l} {\left[ {\prod\limits_{j = 1}^{n} {(1 /\mu_{j} )^{{1 - T_{{a_{j} }}^{L} }} } ,\;\prod\limits_{j = 1}^{n} {(1 /\mu_{j} )^{{1 - T_{{a_{j} }}^{U} }} } } \right],} \hfill \\ {\left[ {1 - \prod\limits_{j = 1}^{n} {(1 /\mu_{j} )^{{I_{{a_{j} }}^{L} }} } ,\;1 - \prod\limits_{j = 1}^{n} {(1 /\mu_{j} )^{{I_{{a_{j} }}^{U} }} } } \right],} \hfill \\ {\left[ {1 - \prod\limits_{j = 1}^{n} {(1 /\mu_{j} )^{{F_{{a_{j} }}^{L} }} } ,\;1 - \prod\limits_{j = 1}^{n} {(1 /\mu_{j} )^{{F_{{a_{j} }}^{U} }} } } \right]} \hfill \\ \end{array} } \right\rangle . $$

Thus, the proof of Eq. (9) is completed. □

Based on the INWEA operator and the exponential operational law of INNs with interval-valued parameters, we can establish another exponential aggregation operator of INNs.

### **Definition 11**

Let $a_{j} = \left\langle {[T_{{a_{j} }}^{L} ,T_{{a_{j} }}^{U} ],[I_{{a_{j} }}^{L} ,I_{{a_{j} }}^{U} ],[F_{{a_{j} }}^{L} ,F_{{a_{j} }}^{U} ]} \right\rangle$ for *j* = 1, 2, …, *n* be a collection of INNs and $\tilde{\mu }_{j} = [\mu_{j}^{L} ,\mu_{j}^{U} ]$ (*j* = 1, 2, …, *n*) be a collection of interval numbers, and let a mapping DINWEA: Ω^*n*^ → Ω. Then, the DINWEA operator is defined as

12 $$ DINWEA(a_{1} ,a_{2} , \ldots ,a_{n} ) = \prod\limits_{j = 1}^{n} {(\tilde{\mu }_{j} )^{{a_{j} }} } , $$

where *a*_*j*_ (*j* = 1, 2, …, *n*) is the exponential weight of $\tilde{\mu }_{j}$ (*j* = 1, 2, …, *n*).

### **Theorem 7**

*Let* $a_{j} = \left\langle {[T_{{a_{j} }}^{L} ,T_{{a_{j} }}^{U} ],[I_{{a_{j} }}^{L} ,I_{{a_{j} }}^{U} ],[F_{{a_{j} }}^{L} ,F_{{a_{j} }}^{U} ]} \right\rangle$ *for j* = 1,2, …, *n be a collection of INNs and* $\tilde{\mu }_{j} = [\mu_{j}^{L} ,\mu_{j}^{U} ]$ (*j* = 1,2, …, *n*) *be a collection of interval numbers, and then the aggregated value of the DINWEA operator is given by*

13 $$ DINWEA(a_{1} ,a_{2} , \ldots ,a_{n} ) = \left\{ {\begin{array}{*{20}l} {\left\{ \begin{aligned} \left\langle \begin{aligned} \left[ {\prod\limits_{j = 1}^{n} {\left( {\mu_{j}^{L} } \right)^{{1 - T_{{a_{j} }}^{L} }} } ,\;\prod\limits_{j = 1}^{n} {\left( {\mu_{j}^{L} } \right)^{{1 - T_{{a_{j} }}^{U} }} } } \right], \hfill \\ \left[ {1 - \prod\limits_{j = 1}^{n} {\left( {\mu_{j}^{L} } \right)^{{I_{{a_{j} }}^{L} }} } ,\;1 - \prod\limits_{j = 1}^{n} {\left( {\mu_{j}^{L} } \right)^{{I_{{a_{j} }}^{U} }} } } \right], \hfill \\ \left[ {1 - \prod\limits_{j = 1}^{n} {\left( {\mu_{j}^{L} } \right)^{{F_{{a_{j} }}^{L} }} } ,\;1 - \prod\limits_{j = 1}^{n} {\left( {\mu_{j}^{L} } \right)^{{F_{{a_{j} }}^{U} }} } } \right] \hfill \\ \end{aligned} \right\rangle , \hfill \\ \left\langle \begin{aligned} \left[ {\prod\limits_{j = 1}^{n} {\left( {\mu_{j}^{U} } \right)^{{1 - T_{{a_{j} }}^{L} }} } ,\;\prod\limits_{j = 1}^{n} {\left( {\mu_{j}^{U} } \right)^{{1 - T_{{a_{j} }}^{U} }} } } \right], \hfill \\ \left[ {1 - \prod\limits_{j = 1}^{n} {\left( {\mu_{j}^{U} } \right)^{{I_{{a_{j} }}^{L} }} } ,\;1 - \prod\limits_{j = 1}^{n} {\left( {\mu_{j}^{U} } \right)^{{I_{{a_{j} }}^{U} }} } } \right], \hfill \\ \left[ {1 - \prod\limits_{j = 1}^{n} {\left( {\mu_{j}^{U} } \right)^{{F_{{a_{j} }}^{L} }} } ,\;1 - \prod\limits_{j = 1}^{n} {\left( {\mu_{j}^{U} } \right)^{{F_{{a_{j} }}^{U} }} } } \right] \hfill \\ \end{aligned} \right\rangle \hfill \\ \end{aligned} \right\},} \hfill & {if\;0 \le \mu_{j}^{L} \le \mu_{j}^{U} \le 1} \hfill \\ {\left\{ \begin{aligned} \left\langle \begin{aligned} \left[ {\prod\limits_{j = 1}^{n} {\left( {1 /\mu_{j}^{U} } \right)^{{1 - T_{{a_{j} }}^{L} }} } ,\;\prod\limits_{j = 1}^{n} {\left( {1 /\mu_{j}^{U} } \right)^{{1 - T_{{a_{j} }}^{U} }} } } \right], \hfill \\ \left[ {1 - \prod\limits_{j = 1}^{n} {\left( {1 /\mu_{j}^{U} } \right)^{{I_{{a_{j} }}^{L} }} } ,\;1 - \prod\limits_{j = 1}^{n} {\left( {1 /\mu_{j}^{U} } \right)^{{I_{{a_{j} }}^{U} }} } } \right], \hfill \\ \left[ {1 - \prod\limits_{j = 1}^{n} {\left( {1 /\mu_{j}^{U} } \right)^{{F_{{a_{j} }}^{L} }} } ,\;1 - \prod\limits_{j = 1}^{n} {\left( {1 /\mu_{j}^{U} } \right)^{{F_{{a_{j} }}^{U} }} } } \right] \hfill \\ \end{aligned} \right\rangle \hfill \\ \left\langle \begin{aligned} \left[ {\prod\limits_{j = 1}^{n} {\left( {1/\mu_{j}^{L} } \right)^{{1 - T_{{a_{j} }}^{L} }} } ,\;\prod\limits_{j = 1}^{n} {\left( {1/\mu_{j}^{L} } \right)^{{1 - T_{{a_{j} }}^{U} }} } } \right], \hfill \\ \left[ {1 - \prod\limits_{j = 1}^{n} {\left( {1 /\mu_{j}^{L} } \right)^{{I_{{a_{j} }}^{L} }} } ,\;1 - \prod\limits_{j = 1}^{n} {\left( {1 /\mu_{j}^{L} } \right)^{{I_{{a_{j} }}^{U} }} } } \right], \hfill \\ \left[ {1 - \prod\limits_{j = 1}^{n} {\left( {1 /\mu_{j}^{L} } \right)^{{F_{{a_{j} }}^{L} }} } ,\;1 - \prod\limits_{j = 1}^{n} {\left( {1 /\mu_{j}^{L} } \right)^{{F_{{a_{j} }}^{U} }} } } \right] \hfill \\ \end{aligned} \right\rangle \hfill \\ \end{aligned} \right\},} \hfill & {if\;\mu_{j}^{U} \ge \mu_{j}^{L} > 1} \hfill \\ \end{array} } \right., $$

*By the similar proof of the INWEA operator in* Theorem 6*, it is obvious that the DINWEA operator in* Theorem 7 *holds for all n and its aggregated value is also a DINN.*

## Decision making methods based on the INWEA and DINWEA operators

Based on the INWEA and DINWEA operators, we can deal with some decision making problems, where the weights of attributes are expressed by INNs and the attribute values are represented by crisp values or interval numbers. Thus, we can establish the decision making methods.

In a MADM problem, assume that *Y* = {*y*_1_, *y*_2_, …, *y*_*m*_} is a set of alternatives and *X* = {*x*_1_, *x*_2_, …, *x*_*n*_} is a set of attributes. Then the suitability judgment of an alternative *Y*_*i*_ (*i* = 1, 2, …, *m*) on an attribute *x*_*j*_ (*j* = 1, 2, …, *n*) is expressed by a crisp value *μ*_*ij*_ ∈ [0, 1] or an interval number $\tilde{\mu }_{ij} = [\mu_{ij}^{L} ,\mu_{ij}^{U} ] \subseteq [0,1]$(*i* = 1, 2, …, *m*; *j* = 1, 2, …, *n*). Thus, we can give an decision matrix *D* = (*μ*_*ij*_)_*m*×*n*_ or $\tilde{D} = \left( {\tilde{\mu }_{ij} } \right)_{m \times n}$. Whereas, the weight of the attribute *x*_*j*_ (*j* = 1, 2, …, *n*) is given by the INN $a_{j} = \left\langle {T_{{a_{j} }}^{{}} ,I_{{a_{j} }}^{{}} ,F_{{a_{j} }}^{{}} } \right\rangle = \left\langle {[T_{{a_{j} }}^{L} ,T_{{a_{j} }}^{U} ],[I_{{a_{j} }}^{L} ,I_{{a_{j} }}^{U} ],[F_{{a_{j} }}^{L} ,F_{{a_{j} }}^{U} ]} \right\rangle$, where $T_{{a_{j} }} \subseteq [0,1]$ indicates the degree that the decision maker prefers to the attribute *x*_*j*_, $I_{{a_{j} }} \subseteq [0,1]$ reveals the indeterminate degree that the decision maker prefers/does not prefer to the attribute *x*_*j*_, and $F_{{a_{j} }} \subseteq [0,1]$ indicates the degree that the decision maker does not prefer to the attribute *x*_*j*_. As for the decision making problem, the steps are described as follows:

- *Step 1*. By using the aggregation operator of Eq. (9) or Eq. (13), we obtain the overall aggregated value *d*_*i*_ = INWEA(*a*_1_, *a*_2_, …, *a*_*n*_) or $\tilde{d}_{i}$ = DINWEA(*a*_1_, *a*_2_, …, *a*_*n*_) (*i* = 1, 2, …, *m*) for each alternative *y*_*i*_ (*i* = 1, 2, …, *m*).
- *Step 2*. According to the measure function of Eq. (5) or Eq. (7), we calculate the measure value of *C*(*d*_*i*_) or $C(\tilde{d}_{i} )$ (*i* = 1, 2, …, *m*).
- *Step 3*. Based on the measure values, we rank the alternatives and select the best one.
- *Step 4*. End.

## Practical example and comparative analysis

### Practical example

This section provides a practical example on the selecting problem of global suppliers to illustrate the application of the proposed decision making methods with crisp values or interval numbers and interval neutrosophic weights.

Some manufacturing company needs to select the best global supplier corresponding to the core competencies of suppliers. The manufacturing company presents a set of four suppliers *Y* = {*y*_1_, *y*_2_, *y*_3_, *y*_4_}, whose core competencies are evaluated by the four attributes: (1) *x*_1_ is the level of technology innovation; (2) *x*_2_ is the degree of reputation; (3) *x*_3_ is the ability of management; (4) *x*_4_ is the level of service. Then, the weight vector for the four attributes is expressed by the INS *N* = {*a*_1_, *a*_2_, *a*_3_, *a*_4_} = {〈[0.6, 0.8], [0.1, 0.3], [0.1, 0.2]〉, 〈[0.6, 0.7], [0.1, 0.3], [0.1, 0.3]〉, 〈[0.6, 0.7], [0.1, 0.3], [0.1, 0.3]〉, 〈[0.7, 0.8], [0.1, 0.3], [0.2, 0.3]〉}, which is given by the decision maker.

Then, the decision maker is required to make the suitability judgment of an alternative *y*_*i*_ (*i* = 1, 2, 3, 4) with respect to an attribute *x*_*j*_ (*j* = 1, 2, 3, 4) and to give the evaluation information of crisp values of *μ*_*ij*_ ∈ [0, 1], which can be structured as the following decision matrix:

$$ D = (\mu_{ij} )_{m \times n} = \left[ {\begin{array}{*{20}c} {0.7} & \quad{0.6} & \quad{0.7} & \quad{0.8} \\ {0.7} & \quad{0.6} & \quad{0.7} & \quad{0.9} \\ {0.7} & \quad{0.7} & \quad{0.8} & \quad{0.8} \\ {0.8} & \quad{0.7} & \quad{0.7} & \quad{0.8} \\ \end{array} } \right]. $$

Then, the proposed decision making method based on the INWEA operator can be applied to solve the selecting problem of suppliers and the decision making steps are described as follows:

- *Step 1*. By using Eq. (9), we calculate the overall aggregated values of attributes for each supplier *y*_*i*_ (*i* = 1, 2, 3, 4):
  When *i* = 1, we can get the following result:
  $$ \begin{aligned} d_{1} = INWEA(a_{1} ,a_{2} ,a_{3} ,a_{4} ) & = \left\langle {\begin{array}{*{20}l} {\left[ {\prod\limits_{j = 1}^{4} {(\mu_{1j} )^{{1 - T_{{a_{j} }}^{L} }} } ,\;\prod\limits_{j = 1}^{4} {(\mu_{1j} )^{{1 - T_{{a_{j} }}^{U} }} } } \right],} \hfill \\ {\left[ {1 - \prod\limits_{j = 1}^{4} {(\mu_{1j} )^{{I_{{a_{j} }}^{L} }} } ,\;1 - \prod\limits_{j = 1}^{4} {(\mu_{1j} )^{{I_{{a_{j} }}^{U} }} } } \right],} \hfill \\ {\left[ {1 - \prod\limits_{j = 1}^{4} {(\mu_{1j} )^{{F_{{a_{j} }}^{L} }} } ,\;1 - \prod\limits_{j = 1}^{4} {(\mu_{1j} )^{{F_{{a_{j} }}^{U} }} } } \right]} \hfill \\ \end{array} } \right\rangle \\ & = \left\langle { [ 0. 5 7 3 1 , { 0} . 6 8 6 5 ] , { [0} . 1 3 4 7 , { 0} . 3 5 2 2 ] , { [0} . 1 5 3 8 , { 0} . 3 2 8 7 ]} \right\rangle \\ \end{aligned} $$
  By the similar calculation, we can obtain the following overall aggregated values:
*d*_2_ = 〈[0.5938, 0.7028], [0.1245, 0.3289], [0.1337, 0.3045]〉 , *d*_3_ = 〈[0.6430, 0.7483], [0.1095, 0.2938], [0.1291, 0.2682]〉, and *d*_4_ = 〈[0.6430, 0.7384], [0.1095, 0.2938], [0.1291, 0.2779]〉.
- *Step 2*. By using Eq. (5), we calculate the measure values of *d*_*i*_ (*i* = 1, 2, 3, 4):
*C*(*d*_1_) = 0.9015, *C*(*d*_2_) = 0.9141, *C*(*d*_3_) = 0.9327, and *C*(*d*_4_) = 0.9308.
- *Step 3*. Since the ranking order of the measure values is *C*(*d*_3_) > *C*(*d*_4_) > *C*(*d*_2_) > *C*(*d*_1_), the ranking order of the four alternatives is *y*_3_ ≻ *y*_4_ ≻ *y*_2_ ≻ *y*_1_. Hence, the alternative *y*_3_ is the best supplier among the four suppliers.

If the suitability judgments of each alternative *y*_*i*_ (*i* = 1, 2, 3, 4) with respect to each attribute *y*_*i*_ (*i* = 1, 2, 3, 4) are represented by the interval numbers of $\tilde{\mu }_{ij} = [\mu_{ij}^{L} ,\mu_{ij}^{U} ] \subseteq [0,1]$(*i* = 1, 2, …, *m*; *j* = 1, 2, …, *n*), which can be structured as the interval-valued decision matrix:

$$ \tilde{D} = (\tilde{\mu }_{ij} )_{m \times n} = \left[ {\begin{array}{*{20}c} {[0.7,0.8]} & {[0.6,0.7]} & {[0.7,0.8]} & {[0.8,0.9]} \\ {[0.8,0.9]} & {[0.6,0.7]} & {[0.7,0.8]} & {[0.9,1.0]} \\ {[0.7,0.8]} & {[0.8,0.9]} & {[0.6,0.8]} & {[0.7,0.8]} \\ {[0.7,0.9]} & {[0.6,0.8]} & {[0.7,0.8]} & {[0.7,0.9]} \\ \end{array} } \right]. $$

In such a decision making problem, the proposed decision making method based on the DINWEA operator can be applied to solve the selecting problem of suppliers and the decision making steps are described as follows:

- *Step 1*. By using Eq. (13), we calculate the overall aggregated values of attributes for each supplier *y*_*i*_ (*i* = 1, 2, 3, 4):
  When *i* = 1, we can obtain the following result:
  $$ \begin{aligned} \tilde{d}_{1} = DINWEA(a_{1} ,a_{2} ,a_{3} ,a_{4} ) & = \left\{ {\begin{array}{*{20}l} {\left\langle {\begin{array}{*{20}l} {\left[ {\prod\limits_{j = 1}^{4} {\left( {\mu_{1j}^{L} } \right)^{{1 - T_{{a_{j} }}^{L} }} } ,\;\prod\limits_{j = 1}^{4} {\left( {\mu_{1j}^{L} } \right)^{{1 - T_{{a_{j} }}^{U} }} } } \right],} \hfill \\ {\left[ {1 - \prod\limits_{j = 1}^{4} {\left( {\mu_{1j}^{L} } \right)^{{I_{{a_{j} }}^{L} }} } ,\;1 - \prod\limits_{j = 1}^{4} {\left( {\mu_{1j}^{L} } \right)^{{I_{{a_{j} }}^{U} }} } } \right],} \hfill \\ {\left[ {1 - \prod\limits_{j = 1}^{4} {\left( {\mu_{1j}^{L} } \right)^{{F_{{a_{j} }}^{L} }} } ,\;1 - \prod\limits_{j = 1}^{4} {\left( {\mu_{1j}^{L} } \right)^{{F_{{a_{j} }}^{U} }} } } \right]} \hfill \\ \end{array} } \right\rangle ,} \hfill \\ {\left\langle {\begin{array}{*{20}l} {\left[ {\prod\limits_{j = 1}^{4} {\left( {\mu_{1j}^{U} } \right)^{{1 - T_{{a_{j} }}^{L} }} } ,\;\prod\limits_{j = 1}^{4} {\left( {\mu_{1j}^{U} } \right)^{{1 - T_{{a_{j} }}^{U} }} } } \right],} \hfill \\ {\left[ {1 - \prod\limits_{j = 1}^{4} {\left( {\mu_{1j}^{U} } \right)^{{I_{{a_{j} }}^{L} }} } ,\;1 - \prod\limits_{j = 1}^{4} {\left( {\mu_{1j}^{U} } \right)^{{I_{{a_{j} }}^{U} }} } } \right],} \hfill \\ {\left[ {1 - \prod\limits_{j = 1}^{4} {\left( {\mu_{1j}^{U} } \right)^{{F_{{a_{j} }}^{L} }} } ,\;1 - \prod\limits_{j = 1}^{4} {\left( {\mu_{1j}^{U} } \right)^{{F_{{a_{j} }}^{U} }} } } \right]} \hfill \\ \end{array} } \right\rangle } \hfill \\ \end{array} } \right\} \\ & = \left\{ \begin{aligned} \left\langle { [ 0. 5 7 3 1 , { 0} . 6 8 6 5 ] , { [0} . 1 3 4 7 , { 0} . 3 5 2 2 ] , { [0} . 1 5 3 8 , { 0} . 3 2 8 7 ]} \right\rangle , \hfill \\ \left\langle { [ 0. 7 0 2 7 , { 0} . 7 8 6 9 ] , { [0} . 0 8 6 8 , { 0} . 2 3 8 5 ] , { [0} . 0 9 6 4 , { 0} . 2 2 1 3 ]} \right\rangle \hfill \\ \end{aligned} \right\}. \\ \end{aligned} $$
  Similarly, we can obtain the following overall aggregated values:
  $$ \begin{aligned} \tilde{d}_{2}   & = \{ \langle [0.6263,\;0.7218],\;[0.1127,\;0.3015],\;[0.1220,\;0.2857]\rangle , \\ & \quad \langle [0.7603,\;0.8228],\;[0.0662,\;0.1858],\;[0.0662,\;0.1772]\rangle \} ; \\ \end{aligned} $$
  $$ \begin{aligned} \tilde{d}_{3}   & = \{ \langle [0.5809,\;0.6957],\;[0.1347,\;0.3522],\;[0.1651,\;0.3287]\rangle , \\ & \quad \langle [0.7501,\;0.8288],\;[0.0746,\;0.2074],\;[0.0950,\;0.1895]\rangle \} ; \\ \end{aligned} $$
  $$ \begin{aligned} \tilde{d}_{4} & = \{ \langle [0.5506,\;0.6684],\;[0.1462,\;0.3777],\;[0.1761,\;0.3551]\rangle , \\ & \quad \langle [0.7770,\;0.8386],\;[0.0636,\;0.1789],\;[0.0734,\;0.1702]\rangle \} . \\ \end{aligned} $$
- *Step 2*. By using Eq. (7), we calculate the measure values of $\tilde{d}_{i}$ (*i* = 1, 2, 3, 4):
*C*($$ \tilde{d}_{1} $$) = 0.9306, *C*($$ \tilde{d}_{2} $$) = 0.9516, *C*($$ \tilde{d}_{3} $$) = 0.9386, and *C*($$ \tilde{d}_{4} $$) = 0.9379.
- *Step 3*. Since the ranking order of the measure values is *C*($\tilde{d}_{2}$) > *C*($\tilde{d}_{3}$) > *C*($\tilde{d}_{4}$) > *C*($\tilde{d}_{1}$), the ranking order of the four alternatives is *y*_2_ ≻ *y*_3_ ≻ *y*_4_ ≻ *y*_1_. Hence, the alternative *y*_2_ is the best supplier among the four suppliers.

### Comparative analysis

For convenient comparison, we use the INWAA operator in the decision making problem with crisp values.

- *Step 1* By using the INWAA operator of Eq. (1), we calculate overall aggregated values of attributes for each supplier *y*_*i*_ (*i* = 1, 2, 3, 4):
  When *i* = 1, we can obtain the following result:
  $$ \begin{aligned} d_{1} ' & = INWAA(a_{1} ,a_{2} ,a_{3} ,a_{4} ) = \sum\limits_{j = 1}^{4} {\mu_{1j} a_{j} = } \left\langle {\begin{array}{*{20}l} {\left[ {1 - \prod\limits_{j = 1}^{n} {\left( {1 - T_{{a_{j} }}^{L} } \right)^{{\mu_{{1_{j} }} }} } ,\;1 - \prod\limits_{j = 1}^{n} {\left( {1 - T_{{a_{j} }}^{U} } \right)^{{\mu_{{1_{j} }} }} } } \right],} \hfill \\ {\left[ {\prod\limits_{j = 1}^{n} {\left( {I_{{a_{j} }}^{L} } \right)^{{\mu_{{1_{j} }} }} } ,\;\prod\limits_{j = 1}^{n} {\left( {I_{{a_{j} }}^{U} } \right)^{{\mu_{{1_{j} }} }} } } \right],\;\left[ {\prod\limits_{j = 1}^{n} {\left( {F_{{a_{j} }}^{L} } \right)^{{\mu_{{1_{j} }} }} } ,\;\prod\limits_{j = 1}^{n} {\left( {F_{{a_{j} }}^{U} } \right)^{{\mu_{{1_{j} }} }} } } \right]} \hfill \\ \end{array} } \right\rangle \\ & = \left\langle { [ 0. 9 3 8 9 ,\; 0. 9 8 1 3 ] ,\; [ 0. 0 0 1 6 ,\; 0. 0 3 4 4 ] ,\; [ 0. 0 0 2 8 ,\; 0. 0 2 5 9 ]} \right\rangle . \\ \end{aligned} $$
  Similarly, we can calculate the overall aggregated values of the attributes for the rest alternatives of *y*_*i*_ (*i* = 2, 3, 4):
*d*_2_′ = 〈[0.9459, 0.9841], [0.0013, 0.0305], [0.0023, 0.0229]〉, *d*_3_′ = 〈[0.9492, 0.9853], [0.0010, 0.0270], [0.0017, 0.0203]〉, and *d*_4_′ = 〈[0.9492, 0.9859], [0.0010, 0.0270], [0.0017, 0.0195]〉.
- *Step 2*. By using Eq. (5), we calculate the measure values of *d*_*i*_’ (*i* = 1, 2, 3, 4):
*C*(*d*_1_′) = 0.9993, *C*(*d*_2_′) = 0.9994 *C*(*d*_3_′) = 0.9995, and *C*(*d*_4_′) = 0.9996.
- *Step 3*. Corresponding to the ranking order of the four measure values *C*(*d*_4_′) > *C*(*d*_3_′) > *C*(*d*_2_′) > *C*(*d*_1_′), the ranking order of the four alternatives is *y*_4_ ≻ *y*_3_ ≻ *y*_2_ ≻ *y*_1_ and the best supplier is *y*_4_.

Thus, we can give the comparative analysis between the INWEA or DINWEA operator and the INWAA operator as follows:

1. In Step 1, the INWEA or DINWEA operator utilizes the attribute weights of the INN *a*_*j*_ and the characteristic value *μ*_*ij*_ ∈ [0, 1] or $\tilde{\mu }_{j} = [\mu_{j}^{L} ,\mu_{j}^{U} ] \subseteq [0,1]$ (*i*, *j* = 1, 2, 3, 4) of an attribute *x*_*j*_ for an alternative *y*_*i*_. However, when we use the INWAA operator, it needs to exchange the roles of *a*_*j*_ and *μ*_*ij*_, i.e., the attribute weight is *μ*_*ij*_ ∈ [0, 1] and the characteristic value of an attribute *x*_*j*_ is *a*_*j*_. Furthermore, the INWA operator cannot deal with the information aggregation operation with the attribute weights of interval numbers. Obviously, the INWAA operator used in these cases is unreasonable; while the INWEA and DINWEA operators used in these cases reveal their rationality because we do not change the meanings and the positions of the weights and the attribute values (crisp values or interval numbers).
2. The two ranking results given by using the INWEA operator and the INWAA operator reveal obvious difference. The main reason is that the positions and meanings of the weights and the attribute values are exchanged respectively, which may result in unreasonable decision making results.

## Conclusion

To extend the existing exponential operations of IVIFSs (Gou et al. 2015b), this paper presented the exponential operational laws of INSs and INNs as a useful supplement of the existing operational laws of INSs and INNs, where the bases are crisp values or interval numbers and the exponents are INSs and INNs. Then, we proposed the INWEA and DINWEA operators with crisp parameters and interval-valued parameters and the comparative methods based on cosine measure functions for INNs and DINNs. Next, we developed the MADM methods based on the INWEA and DINWEA operators. Finally, a practical example was presented to demonstrate the application and rationality of the developed methods. In the future work, the developed methods will be further extended to the neutrosophic overset/underset/offset introduced in (Smarandache 2016) and other fields, such as medical diagnosis, image processing, and clustering analysis.
